# Genome-Wide Identification and Characterization of the DOF Gene Family and Gene Expression Pattern Analysis in Five Legume Species

**DOI:** 10.3390/genes17030324

**Published:** 2026-03-17

**Authors:** Chunyu Nie, Han Zhang, Jiaxin Song, Guohui Xu, Rixin Wang

**Affiliations:** 1 Heilongjiang Provincial Key Laboratory of Oilfield Applied Chemistry and Technology, Daqing 163712, China; 2 College of Bioengineering, Daqing Normal University, Daqing 163712, China; 15146172945@163.com (H.Z.);; 3 College of Bioengineering, Dalian University, Dalian 116622, China

**Keywords:** *legumes*, *DOF* gene family, tissue-specific expression, phylogeny

## Abstract

Background: The DOF transcription factor family is involved in plant growth, development, and stress responses, but systematic comparative genomics studies across legume species are lacking. Methods: We identified the whole genome of the DOF gene family of five legume plants: *Medicago truncatual* (*43*), *Cicer arietinum* (*43*), *Phaseolus vulgaris* (*44*), *Glycine max* (*79)*, and *Lotus japonicus* (*32*). Genome-wide identification of DOF genes was performed in five legume species, followed by phylogenetic analysis, gene structure characterization, duplication event identification, promoter element prediction, synteny analysis, and expression pattern profiling. Results: Phylogenetic comparison with *Arabidopsis thaliana* (47) and *Oryza sativa* (37) classified them into four subfamilies (Groups I–IV). The five legumes all had no more than 30% members of the subgroup. The same subfamily has similar protein structures and gene structures, and most of its members have motif1, with most plants having more than 30% of genes intronic. Gene duplication events were evenly distributed among the members of the DOF gene in all five legumes, and played an important role in its evolution. Moreover, the majority of the DOF genes showed tissue specificity in the five legumes, with most of these members being upregulated in flowers. Additionally, expression pattern analysis under abiotic stress in soybean revealed that members of different subfamilies exhibit divergent expression dynamics under salt, alkali, and cold stresses. The DOF gene family in legumes expanded primarily through segmental duplication and evolved under purifying selection. Conclusion: The subfamily-specific responses to abiotic stress and tissue-specific expression patterns provide candidate gene resources for functional studies aimed at improving stress tolerance and agronomic traits in legume crops.

## 1. Introduction

The DOF (DNA binding with one finger) protein is a plant-specific transcription factor belonging to the zinc finger super-family, and is typically composed of 200–400 amino acids [1]. It possesses two primary domains [2]. Named the DOF domain [3] due to its unique conserved DNA-binding properties, this N-terminal region features a single Cys-rich zinc finger. Specifically, the *DOF* domain contains a C2C2-type zinc finger motif of approximately 50–52 amino acid residues that binds the cis-element 5′-AAAG-3′ [4]. A bipartite nuclear localization signal (NLS), partially overlapping with the DOF domain, is also present. In contrast to the conserved N-terminus, the C-terminal region exhibits high variability and functions as a transcriptional activation domain [5].

DOF proteins play a significant role in regulating diverse plant physiological processes, including growth, development, and responses to both biotic and abiotic stress [6]. Notably, the IF1A protein in *Tamarix chinensis* enhances tolerance to salinity–alkali stress in transgenic plants [7].

With a large number of species among angiosperms, leguminous plants serve as vital sources of starch, oil, and dietary fiber in the human diet. and are major cash and feed crops globally, playing crucial roles in agricultural production [8]. Additionally, they contribute significantly to soil nutrient cycling and ecological preservation. The DOF gene, a plant transcription factor holding a unique evolutionary position, participates in diverse biological processes, including plant growth, carbon and nitrogen metabolism, abiotic stress response, hormone regulation, seed development, and flowering control. In recent years, numerous DOF genes have been identified across various plant species [9,10,11]. For instance, 25, 36, 27, and 28 DOF genes were found in sugarcane, *Arabidopsis thaliana*, poplar, and sorghum, respectively [12,13,14,15]. For this study, we selected 43, 43, 44, 79, and 32 DOF gene families from *Medicago truncatual*, *Cicer arietinum* L., *Phaseolus vulgaris*, *Glycine max*, and *Lotus japonicus*, respectively, for further analysis. They were selected based on a combination of their phylogenetic positions, biological importance, and the availability of high-quality genomic and transcriptomic resources. This selection was designed to ensure a comprehensive and evolutionarily robust analysis of the DOF gene family. Using bioinformatics methods and gene expression analysis, we elucidated the gene sequences, structures, evolutionary relationships, and expression patterns of these leguminous DOF genes across different tissues and under abiotic stress conditions. The findings will facilitate further investigation into the evolutionary relationships and molecular basis of the DOF gene family, providing a theoretical foundation for the functional analysis of DOF proteins under abiotic stress. While genome-wide analyses of the DOF gene family have been conducted in individual legume species such as soybean *Glycine max* [16] and *Medicago truncatula* [17], a systematic comparative genomics study across multiple legume lineages has been lacking. Here, we bridge this gap by performing a unified identification and characterization of DOF genes in five legume species representing different phylogenetic positions and agricultural importance. Our findings not only confirm the expansion of the DOF family via segmental duplications but also reveal differential retention and expression divergence among subfamilies, providing a prioritized list of targets for functional dissection in legumes.

## 2. Materials and Methods

### 2.1. Identification of DOF Genes in Five Legume Species

We obtained the sequence of members of the *Medicago truncatual*, *Cicer arietinum*, *Phaseolus vulgaris*, *Glycine max*, and *Lotus japonicus* DOF family from the PlantTFDB database website (http://planttfdb.gao-lab.org, accessed on 6 August 2021). DOF genes were identified via BLAST searches and hidden Markov model (HMM) analyses. To find candidate *DOF* genes, we performed an extensive search for the DOF domain in five legume plants using the blast search and HMMER v3.3.2 software (http://hmmer.org, accessed on 4 August 2021). Protein molecular weights, isoelectric points, and length for members of each DOF family are predicted through the ExPASy website (https://www.expasy.org/, accessed on 6 May 2022). Detailed information on the DOF protein in five legume plants is shown in Table S1 [18].

### 2.2. Evolution Analysis, Gene Structure, and Conserved Motif Analysis of DOF Genes

The DOF protein sequences identified from the five legume species were integrated with the annotated DOF proteins of *Arabidopsis thaliana* and *Oryza sativa*, and multiple sequence alignment was conducted using ClustalX. A phylogenetic tree was constructed using the neighbor-joining (NJ) method in MEGA 5.0 with 1000 bootstrap replicates. The DOF genes were further divided into different subfamilies based on the homology with five legume plants’ DOF genes in *A. thaliana* and *O. sativa*. By extracting the GFF file information of the five legume plants’ DOF gene family, we obtained the exon, CDS, and UTR position information of the genes on the chromosomes. We conducted a comprehensive analysis of the conserved motifs and gene structures using TBtools v1.098684 software and constructed gene structure diagrams [19].

### 2.3. Chromosome Localization and Gene Duplication Analysis of DOF Genes

We performed sequence comparisons between *Arabidopsis thaliana* and *Oryza sativa* using BLAST v2.12.0 software, and selected gene pairs with more than 75% sequence similarity [20]. The collinearity plot was subsequently constructed using Circos v0.69.9 software [21]. Duplication events were identified based on sequence similarity, alignment coverage, and chromosomal location. All-to-all BLASTP searches were conducted for DOF protein sequences. Tandem duplication events were defined as two or more DOF genes located on the same chromosome within a 100 kb genomic region with no more than one intervening non-homologous gene. Additionally, genomic regions containing three or more DOF genes in close physical proximity (within 200 kb) were defined as gene clusters, regardless of their duplication mechanism. Identified duplication events were further verified using the PDD database (http://chibba.agtec.uga.edu/duplication/, accessed on 4 May 2022). This analysis aimed to elucidate the role of gene duplication in the evolution of the *DOF* gene family.

We identified DOF gene duplication events and performed supplementary verification via the PDD database (http://chibba.agtec.uga.edu/duplication/, accessed on 11 August 2021), two or more DOF genes were considered tandem duplicates if they were located on the same chromosome within a 100 kb genomic region, with no more than one intervening non-homologous gene. Gene clusters were defined as genomic regions containing three or more DOF genes in close physical proximity (within 200 kb), regardless of their duplication mechanism.

### 2.4. Analysis of the Cis-Elements of DOF Promoters

To gain insights into the DOF gene family, we examined the cis-acting regulatory elements in the promoter regions of DOF genes across five legume species.

For promoter analysis, we extracted 1500 bp sequences upstream of the ATG initiation codon and looked for these sequences in the PlantCARE database (http://bioinformatics.psb.ugent.be/webtools/plantcare/html/, accessed on 21 August 2022). The PlantCARE database was used to search for the order elements in the promoter (http://bioinformatics.psb.ugent.be/webtools/plantcare/html/) (accessed on 1 November 2022).

### 2.5. Collinearity Analysis and Ka/Ks Ratio Calculation of the DOF Gene

We employed the MCScanX pipeline to identify potential homologous gene pairs (1 × 10^−5^, top 5 matched pairs). Sequence comparisons between *Glycine max* and *Phaseolus vulgaris*, *Cicer arietinum* and *Glycine max*, *Phaseolus vulgaris* and *Lotus japonicus*, as well as *Medicago truncatual* and *Cicer arietinum* were performed using BLAST. We analyzed the collinearity relationships among the five cultivars.

We used KaKs Calculator 2.0 and the NG method to calculate the non-synonymous substitution rate (Ka) and synonymous substitution rate (Ks) of collinear gene pairs, as well as the Ka/Ks ratio (Table S2).

### 2.6. Analysis of Tissue-Specific Expression and Stress Response Patterns of DOF Genes

The tissue-specific expression patterns of DOF genes, transcriptome data from various tissues of five legume species were obtained from public databases. The data sources were as follows: for *Glycine max* and *Phaseolus vulgaris*, expression data were retrieved from annotation files in the Phytozome v12.1 database; for *Lotus japonicus*, data were obtained from the Lotus Base v3.0 platform (https://lotus.au.dk/expat/, accessed on 1 November 2022). Additionally, raw transcriptome sequencing data for *Cicer arietinum* (accession PRJNA413872) and *Medicago truncatual* (accession PRJNA80163) were downloaded from the NCBI Sequence Read Archive (SRA) database. For the *C. arietinum* and *M. truncatula* datasets, sequencing reads were aligned to their respective reference genomes using TopHat v2.1.0 [22]. Gene expression abundance was quantified and normalized as Fragments Per Kilobase of transcript per Million mapped reads (FPKM) values using Cufflinks v2.1.1 [23] To analyze the expression patterns of DOF genes under abiotic stress conditions in soybean, the GSE57252 dataset was downloaded from the NCBI Gene Expression Omnibus (GEO) database. This dataset includes transcriptome data from *Glycine max* seedlings subjected to salt stress, simulated drought (mannitol treatment), and cold stress treatments. All subsequent analyses were performed independently within each dataset. FPKM values for DOF genes in each species were extracted using Perl scripts. For visualization, FPKM values were log10-transformed, and heatmaps along with K-means clustering were generated using the ggplot2 v3.4.4 package in the R programming environment [24].

### 2.7. Plant Material and Treatments in Five Legume

Our study used plant material and treatments of *Glycine max*. We planted the seeds in a 3:1 (*w*/*w*) mixture of sand and soil, irrigated and watered with 1/2 Hg solution once every 2 days in the greenhouse [25]. Seedlings grow at night at 16–18 °C, 22–24 °C during the day, relative humidity 65–80%, photoperiod 14/10 h (daytime 06:00–20:00), and light intensity 200–230 μmol m^−2^ s^−2^. After 4 weeks, the germinated seedlings were treated with 200 mM NaCl solution (salt: the main causes are osmotic stress and the toxicity of specific ions (Na^+^, Cl^−^)) and 150 mM Na_2_CO_3_ and NaHCO_3_ (saline: simulate alkaline salt stress) or low temperature treatment (cold). The low-temperature treatment was set at 4 °C. The treated seedlings were transferred to an artificial climate chamber set at 4 °C, while other growth conditions were kept consistent with the control conditions. Seedlings were harvested at 0 h (control), 3 h, 6 h, 12 h, and 24 h after the onset of treatment. Treated samples were immediately snap-frozen in liquid nitrogen and stored at −80 °C until RNA extraction.

### 2.8. Synthesis of cDNA, RNA Extraction, and Quantitative Real-Time PCR Analysis

Experimental procedures were performed in accordance with the MIQE guidelines. Total RNA was extracted from the leaves of the entire seedlings using the plant RNA extraction kit. (Tiangen, Beijing, China) following the manufacturer’s instructions. RNA quality was evaluated by electrophoresis on a 1.0% (*w*/*v*) agarose gel stained with ethidium bromide (EB). Genomic DNA contamination was removed by DNase I treatment (Takara, Shiga-ken, Japan). First-strand cDNA was synthesized using a Transcription First Strand cDNA Synthesis Kit (Indianapolis, IN, USA) and served as the template for gene expression analysis. The synthesized cDNA was stored at −80 °C until use. Quantitative real-time polymerase chain reaction (qRT-PCR) was carried out using SYBR Green mix (TransGen Biotech, Beijing, China) on an ABI 7500 Real-Time PCR System (Applied Biosystems, Foster City, CA, USA). Melting curve analysis was performed to verify the specificity of qRT-PCR amplification for the AAAP gene. Expression levels were normalized using the 2^−ΔΔCt^ method with β-actin as the internal reference gene across the five legume species [26]. The primer sequences used for *DOF* gene expression analysis are listed in Table S4.

## 3. Results

### 3.1. Five Legume Species DOF Gene Family Identification and Analysis of Physical and Chemical Properties

We identified and characterized the DOF gene family in five legume species: *Medicago truncatual*, *Cicer arietinum*, *Phaseolus vulgaris*, *Glycine max*, and *Lotus japonicus.* A total of 241 *DOF* genes were identified from the five legume plants (Table S1). The lengths of the protein-coding gene ranged from78 (*GmDOF30*) to 525 (*LjDOF12*) aa, molecular weights ranged from 9156.4 to 58187.38 Da, and isoelectric points ranged from 4.47 (*MtDOF16*) to 10.35 (*MtDOF1*). A total of 162 basic proteins were identified, including *PvDOF9* and *MtDOF19*; a total of 51 acidic proteins were identified, including *CaDOF18* and *PvDOF34*; and a total of 30 neutral proteins were identified, including *CaDOF10* and *MtDOF29.*

### 3.2. Five Legume Species DOF Gene Phylogenetic Tree Analysis

**Figure 1:**
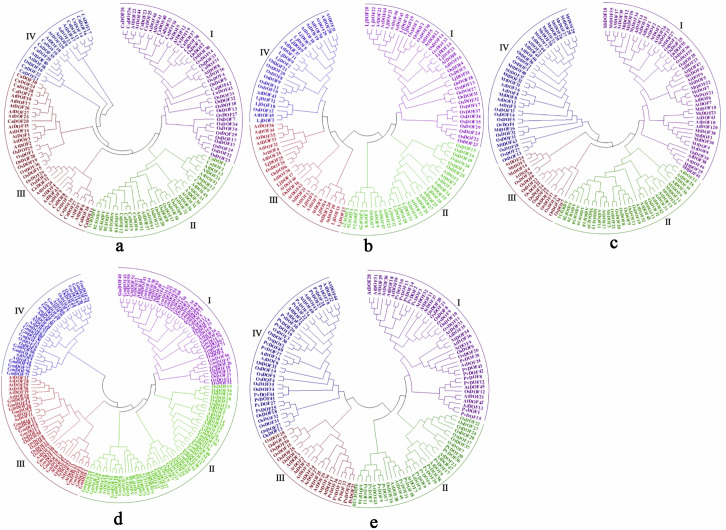
Phylogenetic tree and classification of *DOF* gene families in *Cicer arietinum* (**a**), *Lotus japonicus* (**b**), *Medicago truncatual* (**c**), *Glycine max* (**d**), and *Phaseolus vulgaris* (**e**), subdivided by the following colors: I-purple, II-green, III-red and IV-blue. The phylogenetic tree was constructed using MEGA 5.0 by the neighbor-joining method based on the protein sequences of SBP proteins. The Bootstrap value was 1000 replicates.

We constructed a DOF protein phylogenetic tree using the DOF amino acid sequences of the five legume species, *Arabidopsis thaliana*, and *Oryza sativa* (Figure 1). The phylogenetic tree comprises four major clades: I, II, III, and IV [27]. I and III comprised the highest number of members in *Cicer arietinum*, each containing 13 genes (30.23%), while II exhibited the lowest representation at 18.60%; II comprised the highest number of members in *Lotus japonicus*, each containing 10 genes (31.25%), while III exhibited the lowest representation at 18.75%; and I constituted the largest proportion in *Medicago truncatual* (44.19%), while III represented the smallest fraction (4.65%). I constituted the largest proportion in *Glycine max* (29.11%), while III represented the smallest fraction (20.25%). I constituted the largest proportion in *Phaseolus vulgaris* (31.82%), while III represented the smallest fraction (11.36%). I constituted the predominant group in most species among the five legumes, while III consistently represented the smallest proportion, constituting no more than 30% across these species.

### 3.3. Gene Structure and Motif Analysis of the Five Legume Species DOF Gene

**Figure 2:**
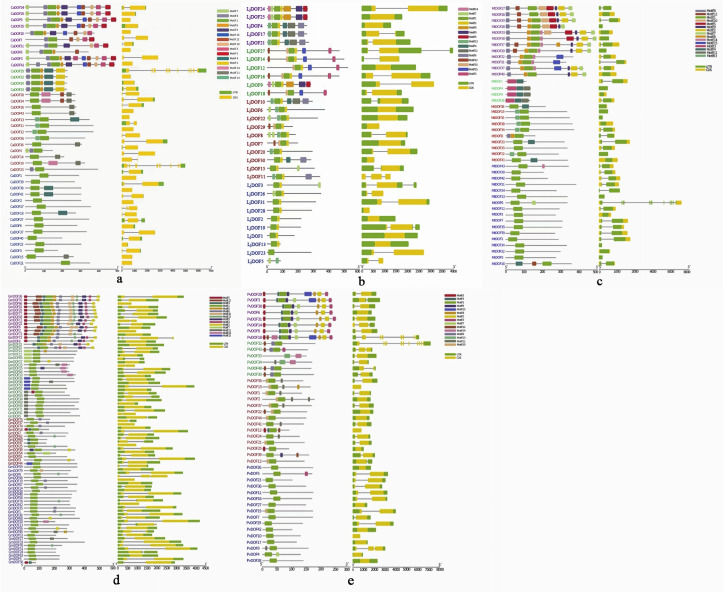
Gene structure analysis of the DOF gene family in the five legumes *Glycine max* (**a**), *Cicer arietinum* (**b**), *Lotus japonicus* (**c**), *Medicago truncatual* (**d**), and *Phaseolus vulgaris* (**e**). Conserved domains of DOF proteins (first column), motif compositions with motif length (second column), and exon–intron structures (third column). Green boxes: introns; yellow boxes: exons; black lines: 5′-3′ untranslated regions (UTRs), subdivided by the following colors: I—purple, II—green, III—red and IV—blue.

The DOF gene family gene structure map and phylogenetic tree with classification (Figure 2) shows that 15 motifs were identified in DOF genes, and motif 1 appeared in almost all DOF genes. All five leguminous plant DOF genes are composed of varying numbers of introns and exons. The number of exons in DOF gene was 1–10.87 DOF genes completely lack introns among the five leguminous plants. Most members exhibited shorter DOF protein sequences with comparatively conserved motif compositions in Subfamily III (*CaDOF6*, *LjDOF1*, *PvDOF6*), all containing Motif 1, while predominantly featuring Motif 8 in *Lotus japonicus.* Most Subfamily II members retain Motifs 3, 4, and 11 in *Medicago truncatual*, with *MtDOF27* and *MtDOF28* having similar distributions of motifs, while *MtDOF5*, *MtDOF10* have similar distributions of motifs. Furthermore, within subfamily III, genes such as *PvDOF6* and *PvDOF8*, along with their homologs in *Phaseolus vulgaris*, exhibited similar motif architectures.

### 3.4. Chromosomal Localization and Gene Replication Relationships

**Figure 3:**
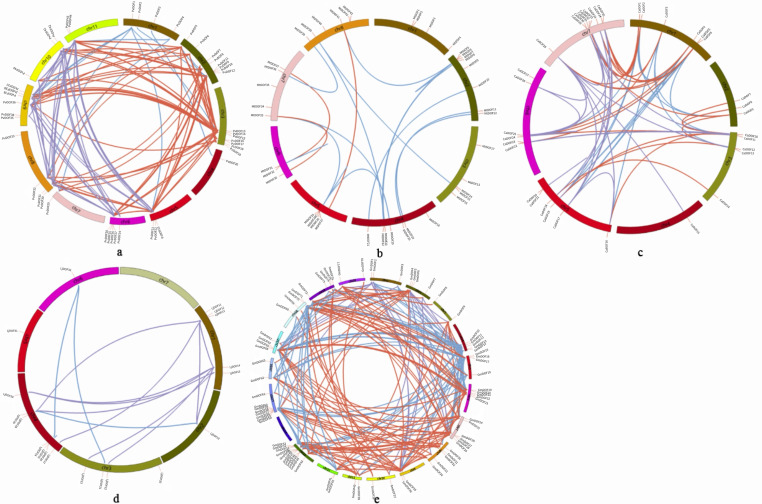
DOF gene family chromosome location and gene replication in *Phaseolus vulgaris* (**a**), *Medicago truncatual* (**b**), *Cicer arietinum* (**c**), *Lotus japonicus* (**d**), and *Glycine max* (**e**). Genes of different subfamilies with gene duplication relationship are connected by different colored lines. Gene clusters are defined as tandemly duplicated gene groups containing three or more genes within a 200 kb interval. Chromosome numbers are indicated at the top of each chromosome.

As shown in the gene duplication map of DOF genes (Figure 3), DOF genes were randomly distributed across all chromosomes, and a total of 241 DOF genes were mapped to chromosomes. Among them, 14 gene pairs were involved in tandem duplication events. Genes were connected with distinct colors to differentiate their similarity indices. Fragment replications events were identified with 125, 17, 12, 226, and 67 pairs in *Phaseolus vulgaris*, *Medicago truncatual*, *Lotus japonicus*, *Glycine max*, and *Cicer arietinum*. Tandem replication events were numbered 3, 4, 1, 3, and 3 pairs, with *Glycine max* exhibiting the highest frequency of duplication events. Among them, the second chromosome of *Phaseolus vulgaris* and *Medicago truncatual* had the largest number of gene members on the chromosome, including eight members. The *Phaseolus vulgaris* highest gene duplication was in chromosomes 2, 3, and 9. The *Medicago truncatual* highest gene duplication was in chromosomes 2 and 4, where there are two gene clusters: *PvDOF13~19*, *PvDOF24~28*, *MtDOF5~9*, *MtDOF25~29.* Chromosome 7 of *Cicer arietinum* harbored the largest number of DOF genes, with nine members. Chromosomes 1, 5, and 7 exhibited the most frequent gene duplication events. Figure 3c shows a single gene cluster designated *CaDOF29~36*. Chromosome 4 of *Lotus japonicus* contained the largest number of gene members, with seven genes. Chromosomes 1 and 4 exhibited the highest levels of gene duplication. Figure 3d shows a single gene cluster designated *LjDOF24~27* and *CaDOF29~36*. Chromosome 13 of *Glycine max* contained the largest number of DOF genes, with 11 members. Chromosomes 8, 13 and 15 showed the highest frequency of gene duplication. Figure 3 contains five gene clusters, designated *GmDOF10~14* and *GmDOF19~23.*

### 3.5. Promoter Element Analysis of the Five Legume Species DOF Gene

The results showed (Table S3) that there are three types of cis-acting elements in the promoters of five leguminous plant DOF genes, which are plant growth and development response element (circadian and HD-zip1), hormone response element (CGTCA-motif, TGACG-motif, ABRE, AuxRR-core, GARE-motif, P-Box and CAT-box), and stress response element (TC-rich repeats, LTR, MBS, ARE, and W box). Among these, ABRE cis-acting elements (abscisic acid-responsive element) were the most abundant, with each *DOF* gene containing 0~4 ABRE elements (Table S3).

### 3.6. Collinearity Analysis of the Five Legume Species DOF Gene

**Figure 4:**
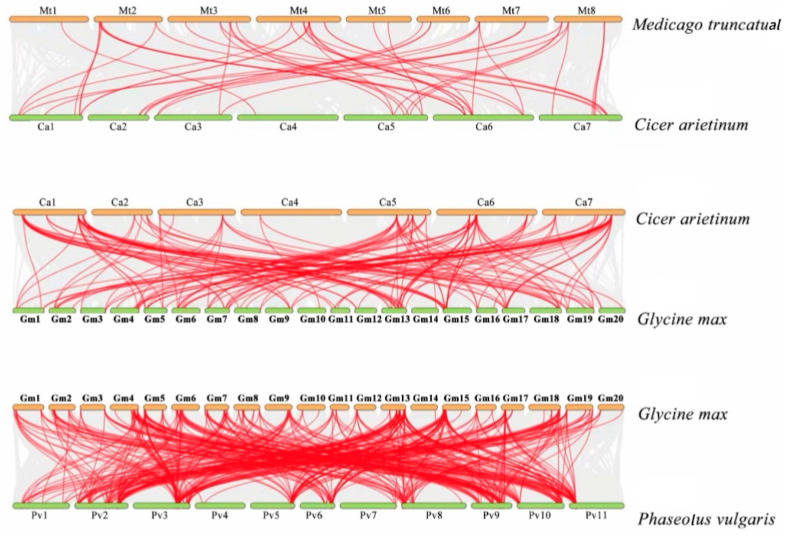
Collinearity analysis of DOF gene family. The grey regions stand for synteny blocks in the whole-genome comparison, and red lines mark collinear gene pairs of the DOF gene family.

Collinearity analysis revealed the distribution of homologous genes between species pairs. To balance visual clarity with the acquisition of key evolutionary insights, we selected five representative species pairs from the 20 possible combinations (Figure 4). The selection was primarily based on phylogenetic relationships and mechanisms of gene expansion: the comparison between *Glycine max* and *Phaseolus vulgaris* was designed to investigate the impact of whole-genome duplication (WGD) on the expansion of the DOF gene family; phylogenetically distant combinations (e.g., *Phaseolus vulgaris* vs. *Lotus japonicus*, and *Cicer arietinum* vs. *Glycine max*) were used to identify orthologous genes under purifying selection; whereas the comparison between closely related species (*Medicago truncatula* vs. *Cicer arietinum*) aimed to analyze chromosomal structural variations following recent speciation events. Specifically, homologous genes of *Glycine max* and *Phaseolus vulgaris* were distributed on 20 and 11 chromosomes, respectively. Between *Cicer arietinum* and *Glycine max*, homologous genes were located on seven and 20 chromosomes, respectively. For *Phaseolus vulgaris* and *Lotus japonicus*, homologous genes were distributed on 11 and 20 chromosomes, respectively. Between *Medicago truncatual* and *Cicer arietinum*, homologous genes were found on eight and seven chromosomes, respectively. Among these, in the chromosomal homology between *Glycine max* and *Phaseolus vulgaris*, *GmDOF13* and *PvDOF9* have the highest homology. In the chromosomal homology between *Cicer arietinum* and *Glycine max*, *CaDOF1* and *GmDOF13* have the highest homology. In the chromosomal homology between *Phaseolus vulgaris* and *Lotus japonicus*, *PvDOF2* and *LjDOF1* have the highest homology. In the chromosomal homology between *Medicago truncatual* and *Lotus japonicus*, *MtDOF4* and *CaDOF6* have the highest relatedness.

To determine the nature and extent of selective pressure by investigating the Darwinian selection relationships of DOF collinear gene pairs across five legume species, the nonsynonymous (Ka) and synonymous (Ks) substitution values were calculated for DOF gene pairs among these species. Generally, a Ka/Ks > 1 is considered indicative of positive selection, whereas Ka/Ks = 1 and Ka/Ks < 1 suggest neutral evolution (pseudogenization) or purifying/negative selection (with a tendency toward purification). As shown in Table S2, the Ka/Ks ratios for DOF genes in the five legume species are all below 1, indicating that *DOF* genes in these legumes have predominantly undergone purifying selection.

### 3.7. Spatiotemporal Expression Profiles of DOF Genes Across Developmental Stages and Tissue Types

**Figure 5:**
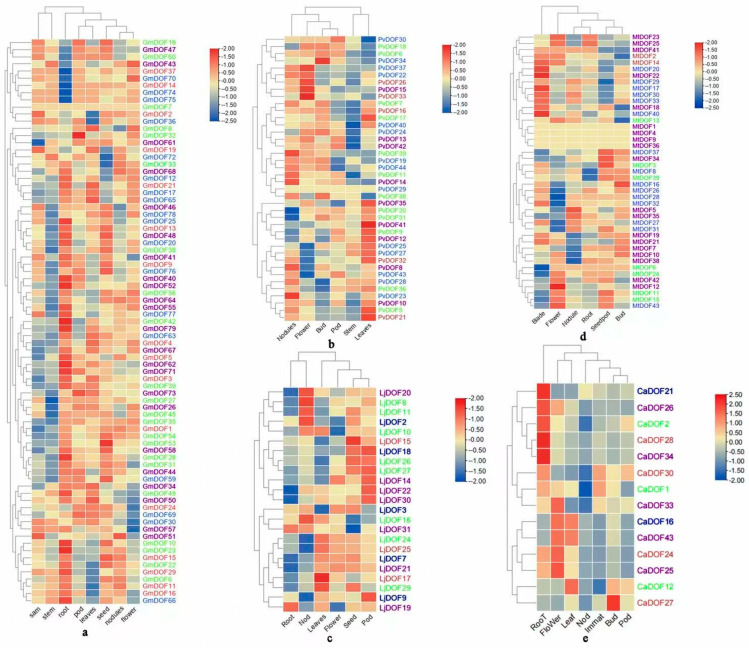
The expression of DOF gene in different tissues of *Glycine max* (**a**), *Phaseolus vulgaris* (**b**), *Lotus japonicus* (**c**), *Medicago truncatual* (**d**), and *Cicer arietinum* (**e**) was analyzed. Only a subset of DOF genes from each species is shown due to space limitations and to highlight genes with clear tissue-specific expression patterns. The selection was based on expression variability across tissues and representation from each phylogenetic subfamily.

The results showed (Figure 5) that in *Medicago truncatual*, the majority of Subfamily I members (e.g., *MtDOF23*) exhibited higher transcript abundance in petals compared to other tissues tested, while the majority of Subfamily IV members (e.g., *MtDOF17)* displayed elevated expression in leaves. In contrast, most genes exhibited relatively low expression levels across all six tissues, with transcript abundances below the average level of the Actin internal control. The results showed that in *Cicer arietinum*, the majority of Subfamily I members (e.g., *CaDOF26*, *CaDOF34*) showed upregulation in root expression, while the majority of Subfamily II members (e.g., *CaDOF1*, *CaDOF2)* displayed downregulation in nodule expression. The results showed that in *Phaseolus vulgaris*, the majority of Subfamily III members (e.g., *PvDOF26*, *PvDOF33)* exhibited upregulation in petal expression, while the majority of Subfamily II members (e.g., *PvDOF6*, *PvDOF18)* displayed downregulation in leaf expression; in *Glycine max*, the majority of members showed upregulation in stem expression, with most displaying downregulation in root expression.

### 3.8. Expression Profile of DOF Genes Under Abiotic Stress in Glycine Max

**Figure 6:**
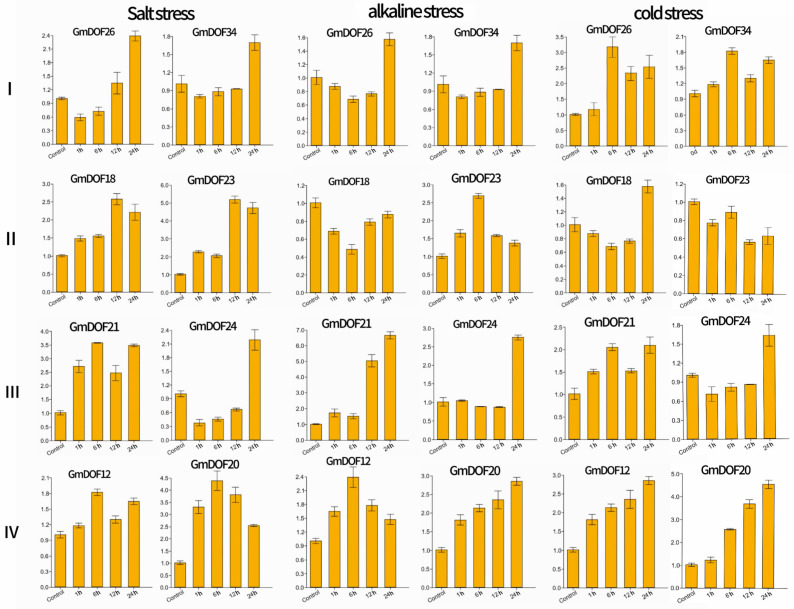
Expression analysis of the DOF genes (qRT-PCR analysis reveals DOF genes under salt, alkali and cold stress in *Glycine max*). The *y*-axis represents the relative gene expression levels calculated using the 2^−ΔΔCt^ method. Expression was normalized to the soybean Actin gene and calibrated to the untreated control (0 h), which was set to 1. I, II, III, IV indicate the subfamilies to which the genes belong.

The expression patterns of two representative genes selected from each *Glycine max* subfamily were analyzed by RT-qPCR under conditions of salt stress, alkali stress, and cold stress (Figure 6 and Figure S1). The genes of subfamily I showed a delayed response, initially declining and then rising, reaching their peaks at 24 h under salt and alkali stress conditions, respectively, indicating a potential role in late-stage stress adaptation. The expression levels of *GmDOF18* and *GmDOF23*, members of subfamily II, display a rapid, transient response, and reached their peak at 12 h, followed by a decline under salt stress conditions. Under alkali stress, the expression levels of subfamily II members peaked at 6 h, followed by a gradual decline, whereas the expression of subfamily IV genes *GmDOF12* and *GmDOF20* reached their highest levels aft er 6 h of salt stress, indicating a specific early response to ionic stress. Under combined stress conditions of salt, alkali, and cold, the expression levels of *GmDOF21* and *GmDOF24*, members of subfamily III, were elevated at 24 h.

## 4. Discussion

A total of 241 DOF genes were identified from the five legume plants, the molecular masses of DOF proteins ranged from 12.10 to 54.41 kDa in *Medicago truncatual*, 16.93 to 54.40 kDa in *Cicer arietinum*, 9.87 to 58.19 kDa in *Lotus japonicus*, 20.77 to 54.79 kDa in *Phaseolus vulgaris*, and 9.16 to 54.67 kDa in *Glycine max*. In *Camelina sativa*, their protein molecular weights range from 19.24 to 63.06 kDa [28]. Protein molecular weights in *Chrysanthemum morifolium* range from 20.78 to 49.67 kDa [4]. This study demonstrates that the molecular weight range of DOF proteins in *Medicago truncatual*, *Cicer arietinum*, *Lotus japonicus*, and *Glycine max* exceeds that weight, being lower than those in *Camelina sativa* and *Chrysanthemum morifolium.*

Gene family expansion results from duplication events occurring either within the entire family or between specific members. In this study, *Phaseolus vulgaris*, *Medicago truncatual*, *Lotus japonicus*, *Glycine max*, and *Cicer arietinum* exhibited 125, 17, 12, 226, and 67 segmental duplication events, and 3, 4, 1, 3, and 3 tandem duplication events. The number of DOF genes in soybean is significantly higher than that in the other four species. This difference is not random, but is closely associated with species-specific genomic evolutionary events. Numerous duplication events have also been identified within the DOF gene family across multiple species. In *Triticum aestivum*, 60 segmental duplication events and three tandem duplication events were detected [25]. In *Brassica napus*, 82 segmental duplication events were identified, while no tandem duplication events were detected [29]. Analysis of *Cerasus humilis* revealed four pairs of tandemly duplicated genes on both Chr5 and Chr8; moreover, gene duplication events played a significant role in the evolution of the DOF gene family in this species [30]. The results indicate that, similar to other species, the expansion of the DOF gene family in legumes occurs primarily through segmental duplication; moreover, gene duplication events likely played a significant role in the evolution of this gene family within legumes. Our analysis revealed 226 segmental duplicate gene pairs in soybean, substantially exceeding those in other species (12–125 pairs), whereas tandem duplications exhibited limited variation across species (1–4 pairs). This suggests that the expansion of the soybean DOF gene family was mainly driven by segmental duplications derived from whole-genome duplication, rather than local tandem duplications. This finding is in agreement with previous research in soybean conducted by Guo et al., which demonstrated that DOF genes were preferentially preserved through segmental duplication [16].

In the present study, 36% of the *AsDOF* genes were found to lack introns. Intronless DOF genes are also widely prevalent in several other species, such as *Brassica napus* (52.14%) [29], *Camelina sativa* (39.8%) [28], and *Chrysanthemum* (20%) [4]. Extensive intron loss within DOF genes is also widely observed across diverse species. In *Brassica napus*, 52.14% of genes were revealed to be intronless, whereas approximately 39.8% of genes in false flax *Camelina sativa* lack introns. In *Chrysanthemum morifolium*, 20% of genes were identified as intronless. Among the plant species investigated in the literature cited herein, extensive gene duplication events are commonly observed. We demonstrate a high frequency of intronless members within this gene family. This can be attributed to the fact that numerous intronless genes participate in gene duplication events.

In this study, it was observed that most members of the DOF subfamily I in five legume species exhibited markedly higher relative expression in roots, with the majority of subfamily I genes in chickpea (*CaDOF26*, *CaDOF34*, etc.) being upregulated in root tissues. In *Arabidopsis*, the homologous transcription factors of *CaDOF26*, *CaDOF34*, and *DOF4.6* interact with *XND1* to coordinately regulate xylem development and aquaporin function in roots [31]. The expression levels of *CaDOF26* and *CaDOF34,* homologs of DOF4.6, were increased in roots compared with the control group (0 h). These results suggest that *CaDOF26* and *CaDOF34* are promising candidates for future functional studies on root development in chickpea (Figure 5). Among the DOF genes in five legume species, *LjDOF19* and *GmDOF3* exhibited distinct differential expression between leaf and root tissues. Enriched with ABA-responsive elements in their promoter regions, their homologous genes *PtrDOF14* and *PtrDOF16* in *Populus tomentosa* showed sustained upregulation under ABA and osmotic stress treatments in both leaf and root tissues [32]. In *Populus tomentosa*, seven genes, including *PtrDOF14* and *PtrDOF16*, exhibited sustained upregulation in both leaf and root tissues under ABA and osmotic stress treatments, suggesting that the legume genes *LjDOF19* and *GmDOF3* may regulate root development through ABA-responsive elements. The expression of *AtDAG1*, a DOF transcription factor regulating germination, was predominantly detected in floral and mature pericarp tissues, with specific localization to the seed coat and phloem [33]. This observation supports the possibility that DOF genes highly expressed in *Medicago truncatual* and *Phaseolus vulgaris petals* (*MtDOF23*, *PvDOF33*, etc.) may also play specific roles in reproductive development, as indicated in the present study. In *Oryza sativa*, *OsDOF6* accumulated predominantly in the leaf sheaths and roots during the vegetative growth phase, with expression peaking in the reproductive stage; its maximal transcript levels were concentrated in the lemma and palea during middle-to-late developmental stages, at levels exceeding those in floral organs and seeds [34]. Our results indicate that *PvDOF36* exhibits specific expression in plant shoots. Consistent with this pattern, its homologous gene in tomato, *SlDOF13*, is expressed exclusively in shoots [35], while potato genes *StDOF29a*, *StDOF32*, and *StDOF34* display enriched expression in shoot tissues compared to other organs [36]. Furthermore, in the study by Guo et al., all members of subgroup VII *GmDOF* genes were also found to be predominantly expressed in shoots [16]. This aligns with the findings of the present study, suggesting that these DOF genes may be involved in the development of specific tissues. This pattern is consistent with our finding that DOF genes in legumes generally exhibit higher expression levels in vegetative organs (roots, stems, leaves) than in reproductive organs. It is worth noting that the tissue types analyzed for gene expression varied among the five legume species due to differences in data availability and tissue sampling strategies across databases. Despite these limitations, we observed that members of specific subfamilies tended to exhibit consistent tissue-specific expression patterns across different species. These findings suggest that, although cross-species comparisons are constrained by heterogeneous tissue coverage, core expression trends associated with subfamily identity remain discernible and biologically meaningful. It is important to acknowledge a limitation in the interpretation of our expression data. The transcript levels presented, derived from RNA and qRT-PCR of whole organs, represent average values across the heterogeneous cell populations within those tissues. This approach cannot resolve cell-type-specific expression patterns, which may be critical for the precise regulatory roles of DOF transcription factors.

Promoter cis-acting element analysis demonstrated that DOF family members are implicated in transcriptional regulation under multiple abiotic stresses (e.g., low temperature and drought), as well as in the signaling pathways of various phytohormones including auxin, methyl jasmonate, abscisic acid, and gibberellin. Specifically, *GmDOF26* exhibited upregulated expression under salt stress, and its promoter region contains auxin-responsive elements, indicating an association between its expression and the auxin signaling pathway. Notably, the number of abscisic acid-responsive elements (ABRE) was particularly prominent, with many previously identified genes associated with plant resistance to abiotic stress containing ABRE. This suggests that DOF genes may influence the expression of salinity tolerance regulatory genes by interacting with ABRE and MBS elements, thereby mediating plant responses to environmental stress [11,12]. Abscisic acid-responsive elements (ABRE) were detected in the promoters of *GmDOF18*, *GmDOF21*, and *GmDOF24.* Consistent with this, these genes all exhibited up-regulated expression under salt stress. In *Cerasus humilis*, the expression of *ChDOF07* and *ChDOF08* decreased under salt stress, potentially linked to their negative correlation with proline and malondialdehyde (MDA) content; contrastingly, the increased expression of the family member *ChDOF11* showed a highly significant positive correlation with MDA content [30]. It is thus speculated that the upregulation of its homologous gene *GmDOF18* under salt stress may play an important role in the salt-stress response by modulating proline and malondialdehyde synthesis (Figure 6). Furthermore, overexpression of the *GhDOFl* gene in *Gossypium hirsutum* upregulates the expression of stress-related genes *GhSOD* and *GhMYB*, thereby enhancing tolerance to both low-temperature and salt stress in cotton [37]. This suggests that its homolog *GmDOF12* may likewise regulate saline–alkali tolerance through modulating genes associated with salt-stress resistance. As a typical member of subgroup III, *GmDOF21* is highly expressed in vegetative organs such as roots and leaves, which aligns with the overall expression pattern of DOF genes in legume plants. Its promoter is enriched with multiple hormone-responsive elements including ABRE, and its expression is upregulated under saline–alkali stress, thereby clearly demonstrating the core mechanism through which DOF genes integrate hormone signaling and stress response to coordinately enhance plant adaptation. This also validates the critical role of DOF genes in both vegetative growth and stress adaptation, as supported by the present study.

## 5. Conclusions

In total, we identified 79, 43, 32, 43 and 44 genes containing DOF from the genomes of *Glycine max*, *Cicer arietinum*, *Lotus corniculatus*, *Medicago truncatual*, and *Phaseolus vulgaris*, respectively. All of the DOF genes can be divided into four groups (I-IV). DOF genes from the same clade have similar motifs and structures, suggesting that the DOF genes from the five legume species may originate from a common ancestor. Furthermore, by comparing five legume species, we revealed the conserved and species-specific mechanisms underlying the evolutionary expansion of the DOF gene family. Selection pressure analysis showed that most of the homologous gene pairs were subject to strong purifying selection of the DOF genes. Most DOF genes exhibit tissue-specific expression patterns, suggesting that they might play key roles in different tissues. DOF genes were involved not only in the regulation of plant growth and development, but also in response to abiotic stresses. Finally, qRT-PCR analysis showed that the DOF genes responded to drought and salt. The results of this study provided a theoretical basis for further studies of DOF gene function. Furthermore, we identified several core candidate genes that may regulate important agronomic traits, such as root development and multiple stress responses, providing valuable targets for subsequent functional studies. The multi-species comparative strategy employed in this study has yielded the following two important advances: precise identification of orthologous genes, allowing the identification of bona fide orthologs through cross-species collinearity analysis, thereby providing a robust foundation for transferring functional knowledge from model species to crops; and facilitation of the detection of lineage-specific patterns in gene structure, promoter composition, and tissue-specific expression that may underlie functional diversification. Furthermore, by integrating evolutionary analysis (phylogeny, synteny, selection pressure) with expression profiling across multiple tissues and stress conditions, this study offers a comprehensive framework for understanding how duplication and divergence have shaped the DOF gene family in legumes. However, it is important to note that the expression patterns reported here are derived from bulk tissue analysis and represent average transcript levels across heterogeneous cell populations. Future studies utilizing single cells will be necessary to resolve cell-type-specific expression and further elucidate the precise regulatory roles of DOF genes in legumes.

## Data Availability

No new data were created or analyzed in this study.

## Supplementary Materials

The following supporting information can be downloaded at: https://www.mdpi.com/article/10.3390/genes17030324/s1, Table S1: List of all DOF genes information identified in the five legume genomes; Table S2: Ka/Ks of DOF genes in five legume species; Table S3: Promoter element of the five legume species DOF gene. Table S4: List of primers used in qRT-PCR. Figure S1: Significant difference analysis of quantitative real-time PCR (qPCR).
