# Supplementary figures and images for: Genome-Wide Identification and Characterization of the DOF Gene Family and Gene Expression Pattern Analysis in Five Legume Species
Source: Genes (Basel). 2026 Mar 17;17(3):324. doi: 10.3390/genes17030324 (PMC13026619; doi:10.3390/genes17030324)

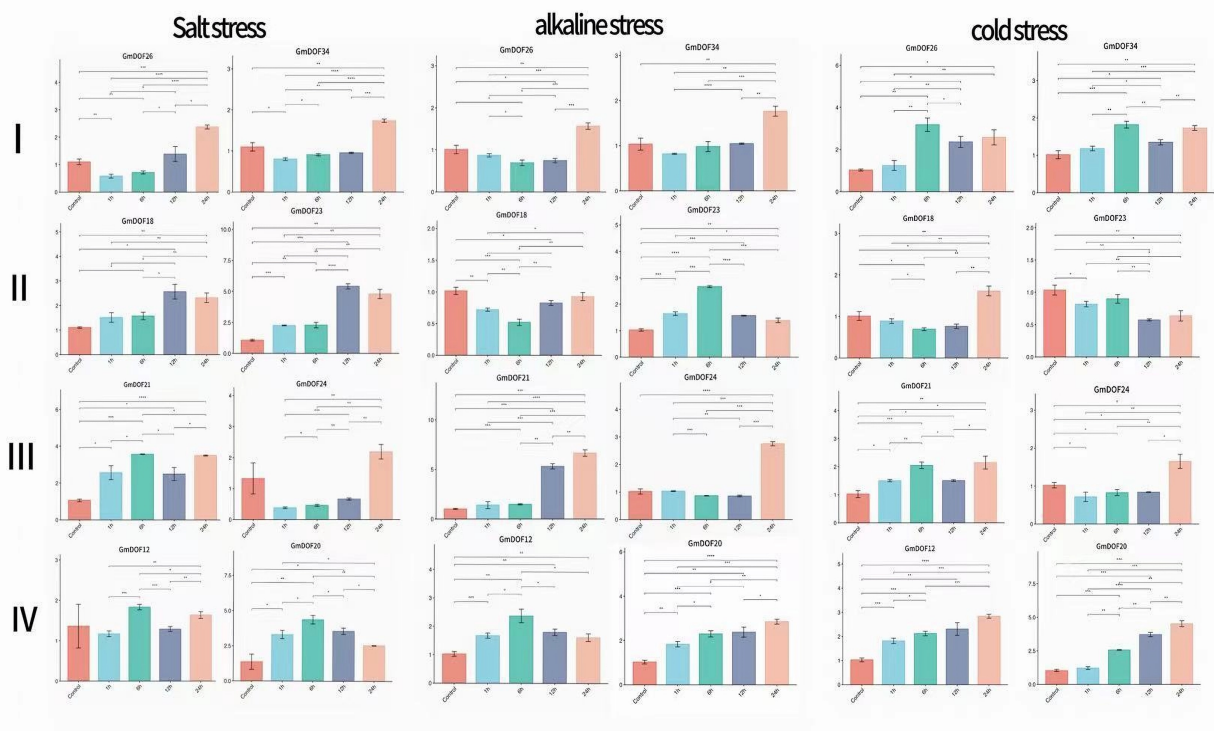

Supplement: Supplementary file 1 [file genes-17-00324-s001.zip › Figure S1.pdf]
